# Teachers’ perceptions of behavioral problems in Dutch primary education pupils: The role of relative age

**DOI:** 10.1371/journal.pone.0204718

**Published:** 2018-10-17

**Authors:** Albert W. Wienen, Laura Batstra, Ernst Thoutenhoofd, Peter de Jonge, Elisabeth H. Bos

**Affiliations:** 1 Department of Developmental Psychology, Faculty of Behavioural and Social Sciences, University of Groningen, Groningen, Netherlands; 2 Department of Special Needs Education and Child Care, Faculty of Behavioural and Social Sciences, University of Groningen, Groningen, Netherlands; 3 Department of Education and Special Education, University of Gothenburg, Gothenburg, Sweden; BC Children’s Hospital Research Institute, CANADA

## Abstract

A growing number of studies suggest that relatively young behavior of pupils gives them a much greater likelihood of being diagnosed with a disorder such as ADHD. This ‘relative age effect’ has also been demonstrated for special educational needs, learning difficulties, being bullied, and so on. The current study investigated the relationship between relative age of pupils in primary education and teachers’ perception of their behavior. The study sample included 1973 pupils, aged between 6 and 12. Six linear mixed models were carried out with birth day in a year as predictor variable and ‘total problem score’, ‘problems with hyperactivity’, ‘behavioral problems’, ‘emotional problems’, ‘problems with peers’ and ‘pro-social behavior’ as dependent variables. Random intercepts were added for school and teacher level. Cluster-mean centering disaggregated between-school effects and within-school effects. We found no associations between relative age of pupils and teacher perceptions of their behavior. Several explanations are postulated to account for these findings which contradict prior studies on relative age effects.

## Introduction

The United Nations (UN) Declaration on the Rights of Persons with Disabilities [1] calls to provide inclusive education at all levels. However, achieving inclusive education is an ongoing challenge in many Western countries [2]. Policy aimed at achieving inclusive education pairs with the wish to more consider what pupils need than what pupils have, also with respect to their behavior in the classroom [3]. However, much special education research nevertheless remains focused on identifying and assessing individual pupils’ dysfunctioning [4], the responsibilities of teachers in diagnosing disorders [5] and the need to identify disorders as early as possible [6].

A growing number of studies suggest that in this signaling function the relatively ‘young behavior’ of early pupils gives them a much greater likelihood of being diagnosed with a disorder, including Attention Deficit Hyperactivity Disorder (ADHD) [7, 8, 9, 10, 11, 12, 13, 14, 15, 16, 17]. This so-called ‘relative age effect’ [18] has also been demonstrated to increase the likelihood of pupils having special educational needs [8, 19], be diagnosed with lower intelligence [20] and learning difficulties [21], attain lower physical education achievements [22, 23, 24], lower performance during the school career [25, 26], and being bullied [27].

In the present study the relationship between relative age and perceived child behavior is investigated in Dutch primary education. We stress that we focus on the perception of teachers, who are asked to judge the behavior of all the pupils in their classroom (one questionnaire for each pupil). Response scores not only tell us something about the behavior of these children but also about the teachers and their judgment approval. Our research question is, ‘What connection is there between the relative age of pupils and the perception of their behavior by their teacher?’ Behavior is separated into hyperactivity, problem behaviors, emotional problems, problems with peers and pro-social behavior. Since girls and boys tend to differ in the kind of (perceived) problems they have, we also investigate possible differences between girls and boys in the influence of relative age on perceived problem behavior [28, 29, 30, 31].

## Method

### Design

This research was performed in an existing data set and falls, in the Dutch situation, outside the scope of the Medical Research Involving Human Subject Act (WMO). No ethical committee approval was requested because this study did not involve medical research. Participants were not subjected to medical procedures or required to follow rules of behavior. Schools informed teachers and parents about the collection of the data and the anonymous transfer of the data to the researchers. The agreements on the use of the data were laid down in an agreement between the schools and researchers.

A cross-sectional survey was conducted involving 29 schools for primary education in Drenthe. Drenthe is a province located in the North-East of the Netherlands. The participating schools were schools who agreed to implement a ‘social and safe school climate’ approach. All 325 schools in Drenthe were contacted in writing to request their participation. Following further contact, 29 regular primary schools chose to implement the social school climate program and join the research. The schools varied with regards to their social economic status, their size, and their denomination (besides public schools, Christian schools are common in the Netherlands). The research was carried out between 2009 and 2015.

### Procedure and respondents

Schools were invited to submit pupil information lists for the classes involved in the study. Teachers completed digital questionnaires about all pupils in their classroom. The birth date, gender and year group of each pupil was recorded. In all, 156 teachers, of 131 classes of 29 primary schools in Drenthe province completed the questionnaires, with a separate questionnaire and login code for each pupil. On average, a teacher completed 12.7 questionnaires (SD = 6.4). An average of 68 questionnaires were completed per school (SD = 42.1). Dutch classrooms sometimes contain combined year groups. In such cases, pupils from more than one year group share a single classroom, so that the age range of pupils in those classrooms is correspondingly wider.

### Independent variable: Birth day

Data were collected in relation to 3372 pupils attending regular schools of primary education in the North of the Netherlands. Pupils who were in special groups for highly gifted children, pupils who were in so-called *schakelklassen* (temporary classrooms between kindergarten and primary school) and pupils of whom it was unclear which year group they were in (which is sometimes the case for pupils in combined year groups), were removed from the data (n = 574). In the Netherlands, most children enter primary school when they are six years old on the first of October. This makes children who were born in September the youngest pupils in class, and children born in October the oldest pupils in class. Within year groups, some pupils were found to be younger than the pupils born in September, for example those who were sent on early into primary education, or pupils who had skipped a year. Likewise, some pupils were found to be older than the pupils born in October, for example those who spent longer time in preschool or who doubled a year. These ‘extremely young’ and ‘extremely old’ pupils (in total n = 825) were removed from the dataset. Thereafter we created the independent variable called ‘birth day’, whereby the youngest pupils, those born on September 30th, were allocated day 1 of the year, while the eldest pupils, born on October 1th, were allocated day 365 of the year (or 366 for leap years). The final study sample included 1973 pupils, aged between 6 and 12, 1008 (51%) boys and 965 (49%) girls, from 29 primary schools in Drenthe province, evaluated by 156 different teachers.

### Dependent variables and measurement instrument

For the measurement of teacher’s perceptions of pupil behavior, the teacher version of the Strengths and Difficulties Questionnaire (SDQ-L) was used. The SDQ was developed on the basis of common child behaviors described in the Diagnostic and Statistical Manual of Mental Disorders [32]. This questionnaire has shown a relatively high reliability [33]. Goedhart, Treffers and Van Widenfelt [34] judged the internal consistency of the questionnaire as ‘good’. A Dutch study [35] concluded that both the internal and external validity of the SDQ-L are between sufficient and good. The SDQ-L includes the following sub-scales: emotional symptoms (range 0–10), behavioral problems (range 0–10), hyperactivity/attention deficit (range 0–10), problems with peers (range 0–10), and pro-social behavior (range 0–10). Each sub-scale consists of five questions and the first four sub-scales collectively comprise the sum scale ‘total problem score’ (range 0–40). All items are scored on a three-point Likert scale with the response options ‘not true’ (0), ‘somewhat true’ (1) and ‘surely true’ (2). Items in the SDQ-L cover behaviors like ‘restless, overly active, can’t sit still for very long’ and ‘rather introvert, tends to play alone’. We calculated reliability scores for the SDQ-L scales. First, the ‘naive’ reliability score was computed, without taking different response levels into account (‘mixed level’). Next, the reliability scores were calculated for the levels of the pupil and the teacher [36]. In Table 1, the reliability scores are listed for both the mixed, teacher and pupil level. The naive reliability scores were all acceptable (>.60) to good (>.80). The reliability at the teacher level was acceptable for emotional problems, total problems, and good for pro-social behavior, but rather low for the other scales. At the pupil level, the reliability for the total problem score and problems with hyperactivity was good, while for the other scales it was acceptable.

**Table 1:** Reliability of the SDQ-L scales for mixed, teacher and pupil levels.

	Mixed level	Level teacher	Level pupil
Emotional problems	.75	.70	.74
Behavioural problems	.72	.49	.67
Problems with hyperactivity	.87	.45	.85
Problems with peers	.68	.52	.62
Pro-social behavior	.79	.84	.72
Total problem score	.85	.63	.81

### Statistical analysis

Six linear mixed models were carried out with birth day as predictor variable and ‘total problem score’, ‘problems with hyperactivity’, ‘behavioral problems’, ‘emotional problems’, ‘problems with peers’ and ‘pro-social behavior’ as dependent variables. Cluster-mean centering was applied to the birth day variable to disaggregate between-school effects and within-school effects [37]. Both the cluster-mean centered variable as well as the cluster means for birth day were included as predictors in the model. Possible interactions between birth day and pupil gender, year group, and combined year group were tested, but removed from the final model if they did not contribute significantly to it. Random intercepts were included in the model at both the school and teacher level. Because the data distributions were skewed, we applied bootstrapping to obtain 95% confidence intervals for the estimates. Bonferroni’s correction was applied to a p-value of 0.05, so that a p-value of (0.05/6) = 0.0083 was used to determine significance.

In order to assess the share of individual teacher variance and school variance against total variance in the different subscales of the SDQ, intraclass correlation coefficients (ICC) were calculated. These are shown in Table 2. The ICC calculations consistently give slightly higher values for teachers, when compared to schools.

**Table 2:** Intraclass correlation coefficients for school and teacher level.

		ICC
Emotional problems	Level school	0.03
	Level teacher	0.11
Behavioural problems	Level school	0.00
	Level teacher	0.21
Problems with hyperactivity	Level school	0.00
	Level teacher	0.06
Problems with peers	Level school	0.02
	Level teacher	0.08
Pro-social behavior	Level school	0.04
	Level teacher	0.18
Total problem score	Level school	0.01
	Level teacher	0.12

## Results

Table 3 shows the linear mixed models results. No significant interaction effects between birth day and pupil gender, year group, and combined year group were found. For all of the outcomes, main effects of birth day were non-significant, both at the between- and the within-school level. Significant main effects were found for gender in nearly all outcomes: teachers reported more pro-social behavior, less behavioral problems, less hyperactivity, less problems with peers and less total problem behaviors for girls. For pro-social behavior a main effect was found for combined year group: combined year groups were associated with higher levels of perceived pro-social behavior.

**Table 3:** Association between relative age and perceived problem behavior in Dutch primary school.

		Estimate	Bootstrapped 95% confidence interval	*P*
Emotional problems	Intercept	-0.02	-1.40 to 1.30	0.97
	Birth day within schools	-0.001	-0.001 to 0.0002	0.18
	Birth day between schools	0.01	-0.01 to 0.02	0.10
	Sex pupil	0.15	0.01 to 0.33	0.06
	Year group	0.05	0.01 to 0.11	0.03
	Combined year group	-0.07	-0.35 to 0.12	0.50
Behavioural problems	Intercept	0.76	-0.45 to 1.89	0.10
	Birth day within schools	0.0001	-0.001 to 0.001	0.80
	Birth day between schools	0.002	-0.007 to 0.01	0.47
	Sex pupil	**-0.67**	**-0.78 to -0.53**	***<* .001**
	Year group	0.01	-0.03 to 0.05	0.74
	Combined year group	0.04	-0.13 to 0.18	0.54
Problems with Hyperactivity	Intercept	**5.54**	**2.91 to 8.21**	***<* .001**
	Birth day within schools	-0.001	-0.02 to 0.0001	0.07
	Birth day between schools	-0.01	-0.03 to 0.01	0.10
	Sex pupil	**-1.62**	**-1.85 to -1.37**	***<* .001**
	Year group	-0.03	-0.09 to 0.06	0.42
	Combined year group	-0.17	-0.46 to 0.09	0.14
Problems with peers	Intercept	1.17	-0.21 to 2.57	0.05
	Birth day within schools	-0.001	-0.001 to 0.0001	0.11
	Birth day between schools	-0.0001	-0.01 to 0.01	0.97
	Sex pupil	**-0.23**	**-0.38 to -0.09**	***<* .001**
	Year group	0.03	-0.00 to 0.09	0.09
	Combined year group	0.02	-0.21 to 0.18	0.87
Pro-social behavior	Intercept	**6.23**	**4.55 to 7.86**	***<* .001**
	Birth day within schools	0.0002	-0.001 to 0.001	0.68
	Birth day between schools	0.001	-0.01 to 0.02	0.14
	Sex pupil	**1.16**	**0.99 to 1.33**	***<* .001**
	Year group	-0.00	-0.07 to 0.04	0.94
	Combined year group	**0.42**	**0.23 to 0.63**	***<* .001**
Total problem score	Intercept	**7.10**	**2.54 to 11.87**	***<* .001**
	Birth day within schools	-0.002	-0.0005 to -0.000001	0.05
	Birth day between schools	-0.002	-0.04 to 0.04	0.88
	Sex pupil	**-2.34**	**-2.80 to -1.82**	***<* .001**
	Year group	0.08	-0.03 to 0.29	0.33
	Combined year group	-0.13	-0.80 to 0.33	0.65

Note. Linear mixed models with bootstrapped confidence intervals. N = 1973

Note. Sex pupil is coded 0 for boys and 1 for girls.

Note. In bold: significant effects after Bonferroni’s correction.

As a sensitivity analysis, we performed the same analysis in the total data set, without excluding the extreme pupils (N = 2798). A significant effect was found for birth day at the within-school level in the model of problems with peers (B = -0.001, 95% CI -0.001 to -0.000, *p* < .001), problems with hyperactivity (B = -0.001, 95% CI -0.002 to -0.000, *p* < .001) and total problem score (B = -0.003, 95% CI -0.005 to -0.001, *p* < .001). The corresponding effect sizes, computed by the formula: B * sd (x) / sd (y), were -0.06 for problems with peers, -0.04 for problems with hyperactivity, and -0.06 for total problem score. According to the definitions of Cohen [38], these effects are very small (‘*small’*: r = 0.10).

## Discussion

In this study of the relationship between relative age and teacher-perceived pupil behavior, effects between schools and effects within schools have been disambiguated because associations at different levels of investigation can be markedly different [37, 39, 40]. After doing so, no effects of relative age were found on perceived emotional problems, behavioral problems, problems with hyperactivity, problems with peers, total problems and pro-social behavior. A sensitivity analysis in the total data set, in which extremely young or old pupils were not removed, showed relative-age effects for problems with peers, problems with hyperactivity and total problem score, although the effect sizes were very small. A limitation of this study was that we did not have information on the representativeness of the selected schools for schools in the Netherlands.

### Relative age: How to interpret contradictory results?

The international literature has demonstrated a relative age effect in relation to outcome measures that range from the likelihood of learning problems [21] to the likelihood of success in playing hockey [41]. For pupil behavior problems, the association between relatively young pupils and the likelihood of receiving an ADHD diagnosis in particular is well documented [9, 10, 11, 12, 13, 14, 15, 16, 17], although some published studies did not find an association [26, 42].

In what follows, we compare our study with the various published studies on behavioral problems. However, our arguments may well apply also to the other perceived behaviors reported in the present study. Various explanations may be proposed for the fact that a relative age effect was not found for perceived emotional, social and behavioral problems in the present study. The first explanation concerns the Dutch school system [26], which is characterized by a large number of special needs education referrals that were made during the last decennia [2]. It might therefore be the case that especially relatively young pupils with high levels of perceived behavioral problems were referred to special education primary schools [19], so that the population pool of our study is biased by their absence.

A second explanation is that in most previously published studies on this topic the likelihood of receiving an ADHD diagnosis and/or medication was investigated, while in the present study a rating list on classroom behavior of pupils was used which was filled out by all teachers. Then again, ADHD is often diagnosed by relying on third party reports (typically by teachers and parents) within the context of a school or home setting, thus highlighting the crucial role that teachers are playing in ADHD diagnosis. Importantly, though, many of the teachers in the present study may have very little or no involvement in suggesting an ADHD diagnosis. Just as a small minority of ADHD prescribers are responsible for most of the ADHD prescriptions [43], a small minority of teachers might be responsible for the majority of teacher-initiated referrals to medical doctors for diagnostic assessments, while the majority of teachers may recognize problem behavior of young children as age-related immaturity or may be more tolerant of varying maturity levels. Unfortunately, our dataset did not enable us to identify differences between teachers who suggest ADHD diagnostic assessments and those who rarely or never suggest an assessment in relatively young children. Future studies may be designed to identify the individual practices of teachers in relation to “suggesting a diagnosis” and then examine whether these moderate the relative-age-dependent perception of problem behavior.

A third explanation may be found in methodological differences. For example, prior studies have not always excluded pupils who doubled and pupils who skipped years from their analyses. However, the markedly different age and school circumstance of these pupils and their likelihood of therein showing different behavior, may confuse the data and cause a potential source of bias in relative age effect findings. Therefore, in the present study, the decision was made to remove extremely young and old pupils from the data, and this decision may have accounted for the difference between our and prior findings. In order to test this supposition, we performed the same analysis in the total data set, i.e. without excluding the extreme pupils (N = 2798). In this sensitivity analysis we found significant relative-age effects for problems with peers, problems with hyperactivity and total problem score, although the effect sizes were very small. Thus, this methodological difference may indeed be one of the explanations for the difference between the present and prior studies.

A final explanation for our negative research findings concerns the specific statistical model used to analyze the data. Whenever research is done on data sets in which the data are clustered, for example because they were collected in different schools [44], in different communities [11], or in different regions [9], it is important to analyze the data using multi-level models in which within- and between-cluster effects are clearly disaggregated and the nesting of measurements in teachers, and teachers in schools, is taken into account. Therefore, in this study, we added random intercepts to the level of the school and the teacher and by cluster-mean centering of the birth day variable. In most of the studies on the relative age effect, this has not been done. It would therefore be interesting to re-analyze the data of these previously published studies using a multi-level approach, in order to gain better insight into the possible association between relative age and behavioral problems.

## Conclusion

The body of evidence demonstrating the relative age effect in the context of ADHD is very large, with studies in many different countries around the world, and with very different methodologies [9, 10, 11, 12, 13, 14, 15, 16, 17]. Hence, on the basis of our relatively small-scale study we cannot conclude that this concerns a spurious rather than a real association. The Dutch school system, characterized by a large number of special needs education referrals that were made during the last decennia, and teacher evaluations on a screening list in the present study as opposed to ADHD diagnosis or medication use as outcome variables in prior research, are more plausible explanations for our findings. Future multi-level studies could focus on heterogeneity across teachers, in order to gain better insight into the association between relative age and behavioral problems, and the role individual teachers play in it.
